# Abstinence-related motivational engagement for smoking cessation: Longitudinal patterns and predictive validity

**DOI:** 10.1371/journal.pone.0247867

**Published:** 2021-03-04

**Authors:** Amanda M. Palmer, Steven K. Sutton, John B. Correa, Vani N. Simmons, Thomas H. Brandon

**Affiliations:** 1 Department of Public Health Sciences, Medical University of South Carolina, Charleston, South Carolina, United States of America; 2 Department of Health Outcomes and Behavior, Moffitt Cancer Center, Tampa, Florida, United States of America; 3 Department of Psychology, University of South Florida, Tampa, Florida, United States of America; 4 Department of Oncologic Sciences, University of South Florida, Tampa, Florida, United States of America; 5 Department of Biostatistics and Bioinformatics, Moffitt Cancer Center, Tampa, Florida, United States of America; 6 Mental Health Service, VA San Diego Healthcare System, San Diego, California, United States of America; 7 Department of Psychiatry, University of California, San Diego, California, United States of America; University of Wisconsin-Madison, UNITED STATES

## Abstract

The Abstinence-Related Motivational Engagement (ARME) scale was developed to assess motivation to remain abstinent after a smoking cessation attempt. The ARME demonstrated reliability and validity among a small sample of ex-smokers. This study expands the psychometric evaluation of the ARME and tests the ARME as a predictor of smoking status among a sample of participants quitting smoking. The parent trial tested the efficacy of a self-help smoking cessation intervention (N = 1874), with assessments every 6 months. Internal consistency and factor structure of the ARME was evaluated at each assessment to confirm use of the measure as designed. Discriminant validity was assessed by comparing the ARME to the Situation-specific Abstinence Self-Efficacy (SSE) scale via inter-correlations and prediction of future smoking status. Finally, the trajectories of both the ARME and SSE were compared among continuous abstainers and continuous smokers. A single-factor structure was observed at each assessment. Cronbach’s alphas ranged from 0.88–0.91 for the total sample. Correlations between the ARME and the SSE ranged from 0.38–0.47 (*p*s <0.001) among smokers; and from 0.09–0.15 (most *p*s > 0.05) among abstainers. Among current smokers, the ARME and SSE were independent positive predictors of subsequent abstinence (AORs 1.28–2.29, *p*s <0.001). For those currently abstinent, only the SSE predicted subsequent abstinence (AORs 1.69–2.60, *p*s <0.05). GEE analyses showed different trajectories for the two measures, as well as between abstainers and smokers. In conclusion, the ARME is a reliable, valid measure with unique predictive utility for current smokers and a distinct trajectory among those who have successfully quit.

## Introduction

Motivation to initiate a smoking cessation attempt and motivation to maintain abstinence can be conceptualized as separate constructs, independently influencing long-term cessation success [1]. A smoking cessation attempt is a dynamic, phasic process [2] characterized by fluctuations in motivation to quit smoking and maintain abstinence. Historically, cessation-related motivation has been assessed at a single time point and often with single-item measures, usually at the initiation of a quit attempt (e.g., Contemplation Ladder [3]). The Abstinence-Related Motivational Engagement scale (ARME) was developed to expand upon assessment of simple desire to quit smoking [1], and further assess abstinence-related engagement throughout the course of a quit attempt. Scale items reflect empirically-derived motivational themes of cognitive effort, priority, vigilance, and excitement.

In a preliminary development and administration study of the ARME, the 16-item version of the scale possessed excellent internal consistency and was associated with current length of smoking abstinence [1]. The ARME was found to have a stronger relationship with length of abstinence than single-item measures of motivation to quit. However, the initial development study had several limitations, including a small sample size and retrospective data collection, thus leaving gaps in the validation of the measure. As a result, the authors encouraged continued exploration and establishment of the psychometric properties of the ARME, and speculated that with this, the ARME scale could be used to predict future abstinence. Measurement of motivational engagement over time could provide insight into the dynamics of the cessation and maintenance processes, which could then be utilized to enhance smoking cessation interventions.

Further evidence for the utility of the ARME has been published in recent years. The ARME has been validated in a Turkish sample, suggesting promise as a robust measure that can be administered internationally [4]. Another study showed a negative correlation between ARME and cessation fatigue, further elucidating the construct [5]. Finally, the ARME was included as an element of a smoking cessation treatment-selecting algorithm for people living with HIV and AIDS [6]. Altogether, this growing body of literature suggests that ARME may be utilized in multiple contexts and across multiple populations to provide insight into smoking cessation and maintenance processes.

The present study evaluated the factor structure, internal consistency reliability, predictive validity, and discriminant validity of the ARME using data from a smoking cessation clinical trial testing a self-help intervention. Assessments included at baseline when all participants were smoking, and five follow-up surveys completed at 6-month intervals following a quit attempt [7]. Based on the development and administration study [1], we hypothesized that, independent of smoking status, the ARME would continue to show a one-factor structure and high internal consistency at all six assessments.

Discriminant validity was evaluated by comparing the ARME to the Situation-Specific Abstinence Self-Efficacy scale [SSE; 8], a measure of self-efficacy in maintaining abstinence. Self-efficacy is a construct that, in theory, increases as mastery of abstinence is achieved. Motivational engagement, as previously discussed, represents a more dynamic trajectory as challenges in quitting and abstaining are experienced. For this reason, we hypothesized that, irrespective of current smoking status, correlations between the two measures would be positive, but relatively small, indicative of discriminant validity.

We also hypothesized that both the ARME and SSE would independently predict subsequent smoking status, based on theory and evidence from the development of the ARME [1].

Predictive validity was evaluated by assessing whether the ARME was associated with subsequent smoking status separately for those currently abstinent and currently smoking. We hypothesized that higher ARME scores would be associated with future abstinence.

Finally, we examined trajectories of the ARME and SSE from baseline to the 30-month assessment for those exhibiting continuous abstinence and for those exhibiting continuous smoking. We hypothesized higher scores among abstaining versus smoking participants at each follow-up for both the ARME and the SSE. However, based on the initial study and the underlying model of SSE, we hypothesized different trajectories for the ARME versus the SSE among long-term abstainers. Specifically, we expected ARME scores to decrease over time as ex-smokers adapt to their abstinence, and SSE scores to increase as continuing abstinence bolstered self-efficacy.

## Methods

Data were collected during a randomized controlled trial of a self-help smoking cessation intervention [7] and are available on Open Science Framework (OSF; doi:10.17605/OSF.IO/6HRZ7). The parent study was approved by the University of South Florida Institutional Review Board, and all participants provided verbal informed consent for participating. At baseline, all participants (N = 1874) were smoking and interested in quitting. After enrollment, all participants received self-help smoking cessation materials (standard care [single booklet], standard intervention [8 booklets over 12 months], or intensive intervention [10 booklets and 9 narrative pamphlets over 18 months]). Participants completed a baseline assessment battery. Follow-up assessments at were delivered at 6, 12, 18, 24, and 30 months.

Of the measures included in each assessment, only the measures used for the present analysis are described. We selected measures similar to those used in the development study [1] to facilitate comparisons between two separate populations (former and current smokers).

### Measures

#### Motivational engagement

The ARME [1] assesses multiple influences involved in motivation to remain abstinent, including cognitive effort (e.g. “I am willing to spend a lot of mental energy on being smoke-free”), priority (e.g. “Being smoke-free is my highest priority at this time”), vigilance (e.g. “I try to anticipate and prepare for any challenges to being smoke-free”), and excitement (e.g. “The thought of being a non-smoker still excites me”). The full measure contains 16 items, each rated on a 7-point Likert scale (1 = completely disagree,” 7 = “completely agree”), and includes four reverse-scored items. The scale ranges from 16 to 112. All items from the full measure can be seen in Table 1.

**Table 1:** ARME scale items and corresponding theme.

Item	Theme
Being smoke-free is my highest priority at this time	Priority
I try to anticipate and prepare for any challenges to being smoke-free	Vigilance
The thought of being a nonsmoker still excites me	Excitement
I spend little time thinking about becoming or staying smoke-free (R)	Cognitive effort
I am doing whatever I can to avoid smoking	Vigilance
I am no longer all that excited about being smoke-free (R)	Excitement
I think about quitting smoking, or staying off cigarettes every single day	Effort
Nothing is more important to me right now than being tobacco free	Priority
I am willing to make sacrifices in other areas in order to be free of cigarettes	Priority
At this time, I am still very excited by the idea of being smoke-free	Excitement
I spend a great deal of time thinking about becoming or staying smoke-free	Cognitive effort
I spend very little time preparing myself for any challenges to being smoke-free (R)	Vigilance
Compared with other things in my life, fighting the urge to smoke is not the top priority for me right now (R)	Priority
I am willing to spend a lot of mental energy on being smoke-free	Cognitive effort
I feel energized just thinking about being smoke-free	Excitement
I am carefully watching out for things that might put me at risk for smoking	Vigilance

(R) indicates reverse-scored item.

#### Self-efficacy

The SSE is a 20-item measure that presents risky situations in which individuals may wish to smoke [8]. The three general categories of situations are Positive/Social (e.g., with friends at a party), Negative/Affective (e.g. when I am extremely anxious and stressed), and Habit/Addictive (e.g. when I first get up in the morning). Participants rated their confidence to avoid smoking for each situation using a 5-point Likert scale (1 = “not at all confident,” 5 = “extremely confident”), producing a scale range from 20 to 100.

#### Smoking status

The primary outcome for the predictive validity analyses was 7-day point prevalence abstinence at each assessment. Those who reported not smoking any cigarettes in the past week were coded as abstinent. Those who reported having smoked one or more cigarettes in the past week were coded as smoking. Those failing to return a survey were coded as missing at that time point.

### Data analysis plan

Response validity was explored through the identification of those with substantial missing data, defined as missing five or more items, or with inconsistent responses to reverse-scored items. Exploratory factor analyses (EFAs) and internal consistency analyses (Cronbach’s α) were conducted to check factor structure and reliability of the ARME as designed. Discriminant validity was evaluated using correlations between the ARME and SSE, and binary logistic regressions were conducted to evaluate predictive validity of the ARME and SSE. Treatment condition was included as a covariate in these regressions, given the significant differences in abstinence rates across conditions at most follow-ups [7]. Finally, generalized estimating equations (GEE) evaluated differences in ARME and SSE trajectories. This analytic approach provides excellent model-based parameter estimates in longitudinal data with numerous missing data patterns. All data processing and analyses were performed using SAS 9.4 (SAS Institute, Cary, NC). Significance was set at *p* = .05 for all statistical tests.

#### Response validity

Table 2 presents ARME sample size at each time point. ARME assessments with five or more missing responses (i.e., > 25%) were removed from the analyses (<2.4% of all returned surveys), and missing responses were imputed using the item mean. Participants who clearly failed to respond appropriately to the four reverse-scored items (e.g., answered all items with either 1’s or 7’s; “straightliners”) were removed from the analyses [9, 10]. This resulted in a loss of less than 4% of data at each time point. Exploratory factor analyses and Cronbach’s alpha were computed using those participants who had no missing responses.

**Table 2:** Sample sizes at each time point.

	Baseline	6-month^1^	12-month	18-month	24-month	30-month
Survey returned	1874	1364	1226	1167	1059	982
Sufficient ARME data^2^	1860	1246	1211	1154	1049	971
Straightliners^3^ (%)	13 (0.7)	24 (1.9)	31 (2.6)	28 (2.4)	34 (3.2)	32 (3.3)
Available for analysis^4^	1847	1222	1180	1126	1015	939
No missing responses	1674	1076	1051	978	889	818

^1^ Due to an error in the structure of the online survey, 90 participants were unable to complete the ARME or SSE at 6 months.

^2^ Twelve (75%) or more of 16 items with a response. Except for the assessment at 6 months, over 98.7% of surveys returned had sufficient ARME data.

^3^ Answered all items with the same response option (e.g., only “1s” or only “7s”).

^4^ Across the six assessments, between 9.7% and 13.1% participants available for analysis had 1–4 missing responses. For those not responding to all items, the range of participants missing a single item was 70–83% and missing two or fewer items was 89–97%. For every survey, every item was missed by at least 1 participant. The maximum number of times an item was skipped was 2.4% (23 of 939) for item 6 of the 30-month survey. The percentage of those not responding to an item was typically higher for the four reverse-scored items.

## Results

### Participant characteristics

Eligibility criteria for enrolling in the parent study were English-speaking adults smoking at least 5 cigarettes per day, reporting a desire to quit smoking (score of “5” or higher on the Contemplation Ladder [3]), and not enrolled in any other quit smoking program [7].

Within the final sample (*N* = 1874) for the clinical trial, mean age was 47.5 years (*SD* = 12.0) and a majority of participants were female (n = 1233; 65.8%). Self-identified races included White (n = 1212; 64.7%), Black/African American (n = 557; 29.7%), and other (n = 94; 5%), with 103 (5.5%) identifying as Hispanic ethnicity. Household income was under $10,000 per year for 612 (32.7%) participants. Mean baseline dependence, as measured by the Fagerström Test of Nicotine Dependence [11], was 5.7 (*SD* = 2.3), indicating moderate nicotine dependence, and mean cigarettes smoked per day upon enrollment was 20.4 (SD = 11.2).

Survey return rates decreased over the 30 months, ranging from 73% at 6 months to 52% at 30 months. Using logistic regression, demographics and baseline smoking-related variables were evaluated separately as predictors of survey return using logistic regression. The following were found to be positive predictors of survey return (ps < .01): women, younger, minority status, and income less than $10,000 per year.

### Factor structure and internal consistency

Because the present sample of current smokers differed from the initial study sample of ex-smokers [1], we used exploratory factor analyses (EFAs) rather than confirmatory factor analyses. Overall, at each assessment time point, three eigenvalues emerged with values greater than 1, a pattern that is consistent with the initial validation study of the ARME [1]. In addition, a large decline from the first to the second eigenvalue was observed at each time point. The first eigenvalue ranged from 6.56 to 7.74 (41.00–48.38% of variance); the second from 1.60 to 1.79 (9.98–11.17% of variance); and the third from 1.15 to 1.29 (7.21–8.09% of variance). Of note, all four reverse-scored items loaded highest on the second factor or had an evenly distributed loading over the 3 factors at all time points.

The large decline from first to second eigenvalue suggests a unidimensional measure, matching the findings from the initial study [1]. The ARME as a unidimensional measure was further supported by the excellent internal consistency at each time point. The range of Cronbach’s α for a one-factor solution of the ARME were 0.88–0.91 for the full sample, 0.89–0.92 among smokers, and 0.84–0.90 among abstainers.

### The ARME and SSE at each assessment

Table 3 presents means and standard deviations for the ARME and SSE, along with ARME-SSE correlation coefficients. Statistics are presented for all responders, for those smoking, and for those abstinent at each assessment.

**Table 3:** Descriptive statistics for Abstinence Related Motivational Engagement (ARME) and Situation-Specific Abstinence Self-Efficacy (SSE) scales at each assessment for all participants and by smoking status.

Assessment	Smoking Status	N ^1^	ARME	SSE ^2^	r	*p*
			*M* (SD)	*M* (SD)		
Baseline	All participants ^3^	1840	84.2 (16.0)	51.7 (17.1)	0.27	< .001
6-month	All participants	1213	82.8 (16.8)	55.7 (19.3)	0.40	< .001
	Smoking	1028	81.2 (16.7)	51.6 (16.4)	0.38	< .001
	Abstinent	185	91.8 (14.3)	78.7 (17.7)	0.12	.100
12-month	All participants	1176	80.6 (17.9)	57.2 (21.2)	0.40	< .001
	Smoking	922	78.5 (17.8)	50.4 (17.0)	0.39	< .001
	Abstinent	254	88.5 (16.0)	82.0 (15.3)	0.11	.068
18-month	All participants	1118	81.0 (17.7)	58.3 (21.6)	0.41	< .001
	Smoking	842	78.6 (17.4)	50.0 (16.2)	0.42	< .001
	Abstinent	276	88.5 (16.5)	83.7 (15.3)	0.09	.119
24-month	All participants	1011	81.0 (18.4)	59.9 (23.1)	0.44	< .001
	Smoking	744	78.3 (18.0)	50.6 (17.4)	0.47	< .001
	Abstinent	267	88.5 (17.4)	86.0 (16.1)	0.15	.019
30-month	All participants	933	81.0 (18.7)	63.1 (23.6)	0.40	< .001
	Smoking	620	78.1 (19.3)	51.3 (17.7)	0.44	< .001
	Abstinent	313	86.7 (16.1)	86.4 (14.7)	0.11	.061

^1^ N is number of participants with ARME score, SSE score, and smoking status. Smoking status reflects self-reported 7-day point prevalence abstinence at each time point.

^2^ The SSE was not evaluated for “straight-liners” as that measure has no reversed-scored items. If 5 or fewer items were missing (≤25%), then item-mean substitution was used.

^3^ All participants were smoking at baseline.

The ARME was modestly correlated (0.27–0.44) with the SSE at all time points for all responders (*p*s <0.001). For those smoking at the follow-up assessments, the correlation of the ARME and SSE ranged from 0.38 to 0.47 (*p*s <0.001). Consistent with the findings in the initial validation study, correlations between the ARME and SSE among those abstinent were uniformly small with only the association at 24 months reaching statistical significance (r = 0.15, p < .05).

### Predicting smoking status

Table 4 presents logistic regression models with either the ARME or the SSE predicting smoking status at the subsequent assessment (e.g., 6 months to 12 months) for those smoking and for those abstinent. The percentage of those changing smoking status ranged from 10.6% to 23.5%, which highlights the dynamic nature of an attempted quit. Prior to analyses, scores on both measures were standardized at each assessment. With standardization, the OR presented is relative to a standard deviation change in the score, which is a more easily interpreted representation of predictive validity. From baseline (all smoking) to 6 months, the ARME was a significant predictor of abstinence (AOR = 1.61, *p* <0.001). That is, each standard deviation of increase in ARME score was associated with 61% greater odds of abstinence. Additionally, the ARME predicted subsequent abstinence for the first 3 follow-ups among smokers (AORs>1.64, *ps* <0.001), suggesting at least a 64% increase in odds of abstinence with each standard deviation increase in ARME score. Among those abstinent, the ARME did not predict future abstinence. In contrast, the SSE was a significant predictor of abstinence at the subsequent follow-up at most time points both for those smoking and for those abstinent (AORs = 1.28–2.60, *ps* <0.05). Of note, OR values and *p*-values were nearly identical when treatment condition was not included in the analyses.

**Table 4:** ARME and SSE (standardized) individually predicting future (subsequent timepoint) abstinence by current smoking status.

	Smoking		ARME^1^		SSE^1^	
Assessment	Status	N	AOR^2^ [95% CI]	*p*	AOR [95% CI]	*p*
Baseline	Abstinent	0	N/A		N/A	
	Smoking	1347	1.61 [1.36, 1.90]	< .001	1.28 [1.11, 1.48]	< .001
6-month	Abstinent	153	0.69 [0.41, 1.15]	.153	1.69 [1.09, 2.59]	.019
	Smoking	843	1.64 [1.31, 2.05]	< .001	1.81 [1.43, 2.30]	< .001
12-month	Abstinent	212	0.65 [0.42, 1.00]	.051	2.60 [1.54, 4.37]	< .001
	Smoking	793	1.70 [1.31, 2.19]	< .001	1.94 [1.47, 2.55]	< .001
18-month	Abstinent	233	0.95 [0.68, 1.32]	.767	2.01 [1.31, 3.11]	.002
	Smoking	703	1.76 [1.36, 2.27]	< .001	2.29 [1.68, 3.13]	< .001
24-month	Abstinent	222	0.72 [0.48, 1.08]	.112	1.89 [1.16, 3.01]	.011
	Smoking	614	1.12 [0.89, 1.41]	.339	1.26 [0.94, 1.70]	.125

^1^ ARME and SSE were standardized by assessment prior to analyses. AOR represents change in odds for a 1 standard deviation increase in the score.

^2^ AOR = Adjusted Odds Ratio, controlling for treatment condition given the main effect of condition of smoking status. Odds ratios were identical to 2 decimal places when analyses were performed without condition as a covariate.

To assess the unique contribution of the ARME and SSE, logistic regressions were performed when each measure independently predicted smoking status. This occurred four times, each of which focused on smokers at the previous assessment. These occurrences coincide with positive correlations between the ARME and SSE, highlighting the importance of multivariable analyses which utilize multiple predictors. At baseline, the ARME remained predictive of abstinence at 6 months (AOR = 1.55 [95% CI 1.30–1.84], *p*<0.001) whereas the SSE did not (AOR = 1.11 [95% CI 0.95–1.29], *p* = 0.195). For those smoking at 6, 12, and 18 months, both the ARME and the SSE remained predictive of abstinence at the subsequent assessment. The three AORs for ARME were 1.39, 1.44, and 1.44 (*p*s<0.01) and for SSE were 1.58, 1.67, and 1.91 (*p*s<0.001).

### Patterns of ARME and SSE among smokers and abstainers

To further differentiate the constructs of abstinence-related motivation and situation-specific self-efficacy, we examined average ARME and SSE scores for two subsets of participants from baseline through 30 months. Those in the ‘abstinent’ group reported abstinence at 3 or more of the 5 follow-up assessments and reported smoking at none of the assessments (n = 117). Those in the ‘smoking’ group reported smoking at 3 or more assessments and abstinence at none (n = 687). Separately for the ARME and SSE, generalized estimating equations (GEE; identity link, AR[1] working correlation matrix) were used to assess main effects for the linear trend for time, the quadratic trend for time, and group (abstinent v. smoking). The interaction term for group with each time variable was included to evaluate potential group differences in ARME and SSE change.

**Fig 1:**
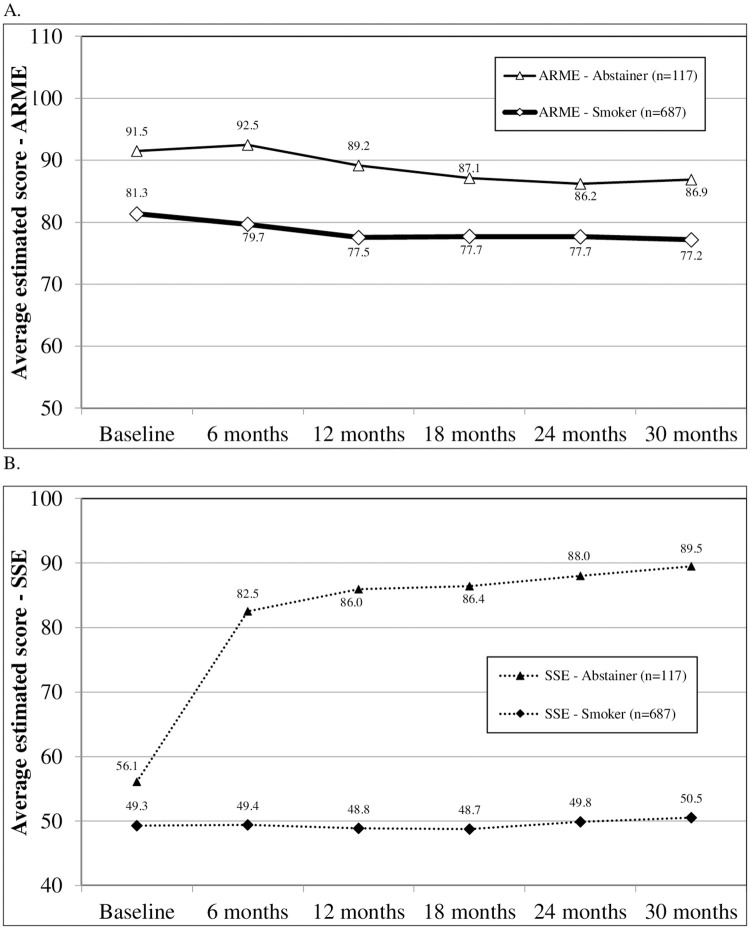
Trajectories for ARME (A) and SSE (B) for those continuously abstinent or continuously smoking during follow up. The ARME scale ranges from 16 to 112. The SSE scale ranges from 20 to 100.

Fig 1a and 1b present average ARME and SSE scores, respectively, by group at each assessment. GEE analysis for average ARME scores revealed no significant interactions (group X linear, group X quadratic), and those terms were dropped from the model. The final model showed three main effects: higher average scores in the abstinent group (χ^2^ [1] = 53.3, *p* < .0001), a negative linear trend (χ^2^ [1] = 34.6, *p* < .0001), and a positive quadratic trend (χ^2^ [1] = 11.1, *p* = .0009). The linear and quadratic trends reflect the overall decrease in average ARME scores over time, independent of smoking status, with slower decrease at later time points.

GEE analysis for average SSE scores found that the abstinent group had higher average scores (χ^2^ [1] = 120.8, p < .0001). The analysis also showed significant linear and quadratic effects for time (χ^2^s>58.3, *p*’s < .0001), and a significant interaction with smoking group (χ^2’^s>68.0, *p*’s < .0001). Simple main effects for the abstinent group showed a significant positive linear and negative quadratic effect for time (χ^2^s>58.0, *p*’s < .0001), which represents the large increase from baseline to 6 months to smaller increase after 6 months. Simple main effects for the smoking group showed a non-significant linear effect and a marginally significant positive quadratic effect (χ^2^ [1] = 58.0, *p* = .052), which represents the relatively flat curve with a slight downward trend to 18 months followed by a slight upward trend to 30 months.

## Discussion

The ARME scale was developed to address a growing interest in the dynamic processes involved in smoking cessation and the role of motivation in these processes [1]. This study further evaluated the ARME, a measure of motivational engagement, through a longitudinal assessment of a sample of smokers enrolled in a smoking cessation trial. Smoking status, the ARME, and the SSE, a measure of cessation self-efficacy, were assessed every 6 months from baseline to 30 months post-enrollment. Our hypotheses were largely supported by the results, which extends findings from the initial cross-sectional validation of the measure. Results also provide insight into the role of motivational engagement during smoking cessation attempts, which can help to inform clinical treatments.

As expected, the ARME demonstrated a stable one-factor structure and high internal consistency at all assessments. The three hypotheses concerning discriminant validity also received support. First, among smokers, the moderate correlations between the ARME and the SSE were significant, but still indicative of discriminant validity. Among abstainers, the small, positive correlations were mostly nonsignificant. The six-month intervals between assessments precluded temporal analyses that that would address important issues. Future research with more frequent assessments, or assessment at well-defined phases of a quit attempt, would allow for calculation of test-retest reliability as well as closer examination of the temporal relationships between these constructs.

Hypotheses concerning predictive validity were partially supported. The ARME predicted abstinence at the subsequent assessment, but only among those currently smoking. In contrast, the SSE predicted abstinence among those currently abstinent and those currently smoking. When predicting future smoking status, multivariable analyses including both ARME and SSE as predictors found the ARME to continue as a significant predictor among current smokers when controlling for the variance due to SSE. Finally, the trajectories of the ARME and SSE averages differed. ARME averages for long-term abstainers were consistently higher than for long-term smokers, with a tendency to decrease over time among both groups. In contrast, SSE averages were initially comparable and relatively low, but the long-term abstainers increased dramatically at 6 months and continued to increase over time. For long-term smokers, SSE averages remained low throughout the study.

### Psychometric implications

These results support three important implications regarding the measurement and assessment of ARME scores. First, abstinence-related motivation can be conceptualized as dynamic during a quit attempt and can be measured as such. The present study supports this conclusion in that average ARME scores differed between those currently abstinent and those currently smoking and changed over the course of a quit attempt. Furthermore, those who exhibited long-term abstinence had a trajectory of average ARME scores that differed from both average ARME scores for long-term smokers and average SSE scores from long-term abstainers.

Second, ARME scores predict changes in smoking status. This study found that the ARME was a stable, strong, positive predictor of future abstinence among those currently smoking. Even when controlling for SSE, a current smoker with an ARME score one standard deviation higher than another current smoker had at least 39% greater odds of being abstinent 6 months later for all but the 24-month assessment. Based on these results, the ARME appears to be a valid measure that can be used in clinical treatment settings and in empirical evaluations of smoking cessation interventions.

Third, these results reinforce that abstinence-related motivation is a separate construct from situation-specific smoking cessation self-efficacy. Throughout the course of the study, elevated ARME scores predicted future abstinence for current smokers. Interestingly, among participants who were abstinent, the ARME did not predict continued abstinence. In contrast, the SSE was predictive of abstinence among *both* smokers and those abstinent. Overall, it appears that both ARME and SSE are important in early stages of cessation. Once one has established abstinence, the ARME loses predictive value for smoking status 6 months later. In contrast, the SSE continues to be a predictor.

Future research could provide further psychometric validation of the ARME as a measure as well as evaluate response differences in various demographic and clinical samples. Aside from test-retest reliability, as noted above, measurement invariance could be evaluated by including the ARME in studies that utilize large, diverse samples. Along similar lines, advanced data analysis techniques could also be applied to future large, longitudinal studies. For instance, Item Response Theory (IRT), Structural Equation Modeling (SEM), and latent modeling techniques could assess item-specific properties, factor structure, and other dynamic features of the ARME as a measure and a construct.

### Implications for smoking cessation

Although the ARME was originally developed to evaluate motivational engagement among individuals who had already achieved smoking abstinence, these results suggest that the clinical utility of the measure is strongest in the early stages of a quit attempt. This has important implications for smoking cessation treatments. For example, if ARME scores are low during contemplation, preparation, and initiation of a quit attempt, interventions should focus on increasing and sustaining motivational engagement (e.g., via motivational interviewing [MI; 12]). This study showed that smokers with higher ARME scores were more likely to be abstinent 6 months later, whereas the ARME was not predictive of abstinence among those currently abstinent. It may be that once a sufficient period of abstinence is achieved, abstinence-related motivation is less critical for future success. Thus, abstinence-related motivational engagement may be a mediating variable that could be targeted as an intermediate outcome toward smoking cessation.

This interpretation is consistent with the observation that abstinence-related motivation changes over time. That is, both successful and unsuccessful quitters showed a drop in ARME scores. Although this may reflect the mastery and automaticity of abstinence-related skills, it is also possible that stressors, triggers, negative affect, and other temptations to smoke could promote the development of “cessation fatigue” [13, 14]. The effort required to maintain abstinence-related motivational engagement may be difficult to sustain for long after a quit attempt. This has implications for personalizing treatments, such as increasing motivational engagement for non-abstainers. Among unsuccessful quitters and relapsers, it may be valuable to re-energize motivational engagement prior to the next quit attempt. Motivational engagement might also influence treatment adherence, including pharmacotherapy (e.g., nicotine replacement therapies), which may in turn impact abstinence rates.

### Limitations

These results should be interpreted within the context of methodological limitations. First, smoking status was determined via self-report and not verified biochemically. Biochemical verification is logistically and financially challenging, especially in nationwide trials like our parent study [15]. Second, the sample size decreased from 1874 from study initiation at baseline, to 982 participants at the 30-month follow-up, reflecting a 52% response rate at the final assessment. Although the final sample size was still quite large, missing data will restrict generalizability of the results. Indeed, having several demographic predictors of survey return challenge the assumption of missing completely at random and suggests the missing at random assumption is plausible. Third, the 6-month assessment intervals made it impossible to quantify momentary, temporal relationships across the ARME, the SSE, and smoking status. Finally, this study was a secondary analysis; as such, measures utilized were limited to those selected for the initial parent RCT. For instance, additional measures of abstinence-related expectancies [16], cessation fatigue [13], or negative affect [17] could be helpful to examine implications of changes in ARME scores over time. This would also provide further opportunities to establish discriminant and predictive validity of these scales.

Results also suggest that the ARME may have limited capabilities in predicting relapse among those who are abstinent. In the present analysis, the ARME was a significant predictor of abstinence among those smoking, which suggests clinical utility of this measure with smokers. However, among abstainers, it may be difficult to identify those at risk for relapse to smoking using the ARME alone. As previously mentioned, perhaps more advanced psychometric evaluation techniques, such as IRT and latent modeling, could provide insight into response-specific indicators of relapse among those who have achieved abstinence. Finally, because of the nature of the parent trial, there was significant missing data among the full sample at each follow-up assessment, which may limit the robustness of the results reported herein.

## Conclusions

Our results indicate that the ARME is a reliable, valid, and psychometrically sound measure of abstinence-related motivational engagement among both smokers and successful abstainers. Although the ARME was correlated with a traditional measure of smoking cessation self-efficacy among smokers, abstinence-related motivational engagement appears to be independent of self-efficacy, with unique predictive power on future abstinence among smokers prior to cessation or after relapse. Enhancement of motivational engagement might be especially useful for smokers preparing to make or to sustain a quit attempt. Future studies should evaluate whether motivational engagement can be enhanced by focused interventions, and if this enhancement produces superior long-term abstinence or treatment adherence outcomes. Finally, the construct of abstinence-related motivational engagement should be evaluated among users of alternative nicotine and tobacco products (e.g., electronic cigarettes, hookah) and users of other potentially addictive substances (e.g., alcohol, cannabis).

## References

[1] Simmons VN, Heckman BW, Ditre JW, Brandon TH. A measure of smoking abstinence-related motivational engagement: Development and initial validation. Nicotine & Tobacco Research. 2010;12(4):432–7. 10.1093/ntr/ntq020 20190004 PMC2847080

[2] Piasecki TM, Fiore MC, McCarthy DE, Baker TB. Have we lost our way? The need for dynamic formulations of smoking relapse proneness. Addiction. 2002;97(9):1093–108. 10.1046/j.1360-0443.2002.00216.x 12199822

[3] Biener L, Abrams DB. The Contemplation Ladder: Validation of a measure of readiness to consider smoking cessation. Health Psychology. 1991;10(5):360–5. 10.1037//0278-6133.10.5.360 1935872

[4] Yavan T, Gulesen A, Bebis H. Abstinence-Related Motivational Engagement Scale: Validity and Reliability in Turkish People. Turkish Thoracic Journal. 2018;19(4):176–81. 10.5152/TurkThoracJ.2018.17100 30322436 PMC6196906

[5] Mathew AR, Heckman BW, Meier E, Carpenter MJ. Development and initial validation of a cessation fatigue scale. Drug and Alcohol Dependence. 2017;176:102–8. 10.1016/j.drugalcdep.2017.01.047 28531766 PMC5802379

[6] Cropsey KL, Bean MC, Haynes L, Carpenter MJ, Richey LE. Delivery and implementation of an algorithm for smoking cessation treatment for people living with HIV and AIDS. AIDS Care. 2019;32(2):223–9. 10.1080/09540121.2019.1626340 31174425 PMC7581129

[7] Brandon TH, Simmons VN, Sutton SK, Unrod M, Harrell PT, Meade CD, et al. Extended Self-Help for Smoking Cessation. American Journal of Preventive Medicine. 2016;51(1):54–62. 10.1016/j.amepre.2015.12.016 26868284 PMC4914420

[8] Velicer WF, Diclemente CC, Rossi JS, Prochaska JO. Relapse situations and self-efficacy: An integrative model. Addictive Behaviors. 1990;15(3):271–83. 10.1016/0306-4603(90)90070-e 2378287

[9] Schonlau M, Toepoel V. Straightlining in Web survey panels over time. Survey Research Methods. 2015;9(2). 10.18148/srm/2015.v9i2.6128

[10] Lugtig P, Toepoel V, Amin A. Mobile-only web survey respondents. Survey Practice. 2016;9(4):1–8. 10.29115/sp-2016-0020

[11] Heatherton TF, Kozlowski LT, Frecker RC, Fagerstrom K-O. The Fagerstrom Test for Nicotine Dependence: a revision of the Fagerstrom Tolerance Questionnaire. Addiction. 1991;86(9):1119–27. 10.1111/j.1360-0443.1991.tb01879.x 1932883

[12] Rollnick S, Miller WR. What is Motivational Interviewing? Behavioural and Cognitive Psychotherapy. 1995;23(4):325–34. 10.1017/s135246580001643x 19364414

[13] Heckman BW, Mathew AR, Carpenter MJ. Treatment burden and treatment fatigue as barriers to health. Current Opinion in Psychology. 2015;5:31–6. 10.1016/j.copsyc.2015.03.004 26086031 PMC4465180

[14] Liu X, Li R, Lanza ST, Vasilenko SA, Piper M. Understanding the role of cessation fatigue in the smoking cessation process. Drug and Alcohol Dependence. 2013;133(2):548–55. 10.1016/j.drugalcdep.2013.07.025 23954071 PMC4057045

[15] Benowitz NL, Bernert JT, Foulds J, Hecht SS, Jacob P, Jarvis MJ, et al. Biochemical Verification of Tobacco Use and Abstinence: 2019 Update. Nicotine Tob Res. 2020;22(7):1086–97. Epub 2019/10/02. 10.1093/ntr/ntz132 . 31570931 PMC7882145

[16] Hendricks PS, Wood SB, Baker MR, Delucchi KL, Hall SM. The Smoking Abstinence Questionnaire: measurement of smokers’ abstinence-related expectancies. Addiction. 2011;106(4):716–28. 10.1111/j.1360-0443.2010.03338.x 21205053 PMC3348861

[17] Brandon TH. Negative Affect as Motivation to Smoke. Current Directions in Psychological Science. 1994;3(2):33–7. 10.1111/1467-8721.ep10769919

